# Gestational weight gain outside the Institute of Medicine recommendations and adverse pregnancy outcomes: analysis using individual participant data from randomised trials

**DOI:** 10.1186/s12884-019-2472-7

**Published:** 2019-09-02

**Authors:** Ewelina Rogozińska, Javier Zamora, Nadine Marlin, Ana Pilar Betrán, Arne Astrup, Annick Bogaerts, Jose G. Cecatti, Jodie M. Dodd, Fabio Facchinetti, Nina R. W. Geiker, Lene A. H. Haakstad, Hans Hauner, Dorte M. Jensen, Tarja I. Kinnunen, Ben W. J. Mol, Julie Owens, Suzanne Phelan, Kristina M. Renault, Kjell Å. Salvesen, Alexis Shub, Fernanda G. Surita, Signe N. Stafne, Helena Teede, Mireille N. M. van Poppel, Christina A. Vinter, Khalid S. Khan, Shakila Thangaratinam, for the International Weight Management in Pregnancy (i-WIP) Collaborative Group, Arne Astrup, Ruben C. Barakat, Annick Bogaerts, Jose G. Cecatti, Jodie M. Dodd, Arri Coomarasamy, Roland Devlieger, Nermean El Beltagy, Fabio Facchinetti, Nina R. W. Geiker, Kym Guelfi, Lene A. H. Haakstad, Cheryce Harrison, Hans Hauner, Dorte M. Jensen, Tarja I. Kinnunen, Khalid S. Khan, Janette Khoury, Riitta Luoto, Ben W. Mol, Siv Mørkved, Narges Motahari, Fionnuala McAuliffe, Julie Owens, Maria Perales, Elisabetta Petrella, Suzanne Phelan, Lucilla Poston, Mireille van Poppel, Kathrin Rauh, Kristina M. Renault, Ewelina Rogozińska, Linda R. Sagedal, Kjell A. Salvesen, Tânia T. Scudeller, Gary X. Shen, Alexis Shub, Signe N. Stafne, Fernanda Surita, Helena Teede, Shakila Thangaratinam, Serena Tonstad, Christina A. Vinter, Ingvild Vistad, Marcia Vitolo, Seonae Yeo

**Affiliations:** 1 Meta-Analysis Group, MRC Clinical Trials Unit at UCL, Institute of Clinical Trials & Methodology, 90 High Holborn, 2nd Floor, London, WC1V 6LJ UK; 2 Women’s Health Research Unit, Barts and the London School of Medicine and Dentistry, Queen Mary University of London, London, UK; 3 Clinical Biostatistics Unit, Hospital Ramon y Cajal (IRYCIS) CIBER Epidemiology and Public Health, Madrid, Spain; 4 Pragmatic Clinical Trials Unit, Barts and the London School of Medicine and Dentistry, Queen Mary University of London, London, UK; 5 Department of Reproductive Health and Research, World Health Organization, Avenue Appia 20, 1211 Geneva, Switzerland; 6 Department of Nutrition, Exercise and Sports, University of Copenhagen, Nørre Allé 51, DK-2200 Copenhagen, Denmark; 7 Department of Development and Regeneration, KU Leuven, Herestraat 49 - Box 805, B-3000 Leuven, Belgium; 8 Faculty of Medicine and Health Sciences, Centre for Research and Innovation in Care (CRIC), University of Antwerp, Antwerp, Belgium; 9 Rua Tessália Vieira de Camargo, 126 Cidade Universitária Zeferino Vaz, São Paulo, Campinas CEP, 13083-887 Brazil; 10 Department of Obstetrics and Gynecology, School of Medical Sciences, University of Campinas, Campinas, Brazil; 11 Women’s and Children’s Hospital, Women’s and Children’s Health Network, Women’s and Babies Division, 72 King William St, North Adelaide, SA 5006 Australia; 12 The Robinson Research Institute, School of Medicine, Department of Obstetrics and Gynaecology, University of Adelaide, Norwich Centre, 55 King William St, North Adelaide, SA 5006 Australia; 13 Obstetrics and Gynecology Unit, Mother Infant Department, University of Modena and Reggio Emilia, largo del Pozzo 71, 41124 Modena, Italy; 14 Clinical Nutrition Research Unit, Copenhagen University Hospital Gentofte, Kildegårdsvej 28, DK-2900 Hellerup, Copenhagen, Denmark; 15 Department of Sports Medicine, Norwegian School of Sports Sciences, Sognsveien 220, 0863 Oslo, Norway; 16 Else Kröner-Fresenius-Zentrum für Ernährungsmedizin, Klinikum rechts der Isar, Technical University of Munich, Georg-Brauchle-Ring 62, 80992 Munich, Germany; 17 Steno Diabetes Center Odense and Department of Gynaecology and Obstetrics, Odense University Hospital, University of Southern Denmark, Kløvervænget 6/4, 5000 Odense, Denmark; 18 Department of Clinical Research, Faculty of Health Sciences, University of Southern Denmark, Odense, Denmark; 19 Unit of Health Sciences, Faculty of Social Sciences, University of Tampere, 33014 Tampere, Finland; 20 Department of Obstetrics and Gynaecology, Nursing and Health Sciences, Monash University, Melbourne, Victoria 3800 Australia; 21 Deputy Vice-Chancellor Research Office, Deakin University, Geelong, Australia; 22 Kinesiology Department, California Polytechnic State University, 1 Grand Avenue, San Luis Obispo, CA 93407 USA; 23 Department of Obstetrics and Gynaecology, Copenhagen University Hospital Hvidovre, Kettegård Alle 30, 2650 Hvidovre, Denmark; 24 Obstetric Clinic, JMC, Copenhagen University Hospital Rigshospitalet, Copenhagen, Denmark; 25 Department of Laboratory Medicine Children’s and Women’s Health, Faculty of Medicine, Norwegian University of Science and Technology, Olav Kyrres gate 11, 7006 Trondheim, Norway; 26 Department of Obstetrics and Gynaecology, St. Olavs Hospital, Trondheim University Hospital, Trondheim, Norway; 27 Department of Obstetrics and Gynaecology, University of Melbourne, Melbourne, Victoria 3010 Australia; 28 Department of Perinatal Medicine, Mercy Hospital for Women, Postboks 8905, N-7491 Trondheim, Norway; 29 Department of Public Health and Nursing, Faculty of Medicine and Health Sciences, Norwegian University of Science and Technology, Trondheim, Norway; 30 Department of Clinical Service, St. Olavs Hospital, Trondheim University Hospital, Trondheim, Norway; 31 Monash Centre for Health Research and Implementation, School of Public Health, Monash University and Monash Health, 246 Clayton Rd, Clayton, VIC 3124 Australia; 32 Institute of Sports Science, University of Graz, Mozartgasse 14,, 8010 Graz, Austria; 33 Department of Public and Occupational Health, EMGO Institute for Health and Care Research, VU University Medical Center, Amsterdam, Netherlands; 34 Department of Gynaecology and Obstetrics, Odense University Hospital, University of Southern Denmark, Sdr. Boulevard 29, DK-5000 Odense, Denmark; 35 Multidisciplinary Evidence Synthesis Hub, Barts and the London School of Medicine and Dentistry, Queen Mary University of London, London, UK

**Keywords:** Gestational weight gain, Body mass index, Institute of Medicine, Individual participant data

## Abstract

**Background:**

High Body Mass Index (BMI) and gestational weight gain (GWG) affect an increasing number of pregnancies. The Institute of Medicine (IOM) has issued recommendations on the optimal GWG for women according to their pre-pregnancy BMI (healthy, overweight or obese). It has been shown that pregnant women rarely met the recommendations; however, it is unclear by how much. Previous studies also adjusted the analyses for various women’s characteristics making their comparison challenging.

**Methods:**

We analysed individual participant data (IPD) of healthy women with a singleton pregnancy and a BMI of 18.5 kg/m^2^ or more from the control arms of 36 randomised trials (16 countries). Adjusted odds ratios (aOR) and 95% confidence intervals (CI) were used to describe the association between GWG outside (above or below) the IOM recommendations (2009) and risks of caesarean section, preterm birth, and large or small for gestational age (LGA or SGA) infants. The association was examined overall, within the BMI categories and by quartile of GWG departure from the IOM recommendations. We obtained aOR using mixed-effects logistic regression, accounting for the within-study clustering and a priori identified characteristics.

**Results:**

Out of 4429 women (from 33 trials) meeting the inclusion criteria, two thirds gained weight outside the IOM recommendations (1646 above; 1291 below). The median GWG outside the IOM recommendations was 3.1 kg above and 2.7 kg below. In comparison to GWG within the IOM recommendations, GWG above was associated with increased odds of caesarean section (aOR 1.50; 95%CI 1.25, 1.80), LGA (2.00; 1.58, 2.54), and reduced odds of SGA (0.66; 0.50, 0.87); no significant effect on preterm birth was detected. The relationship between GWG below the IOM recommendation and caesarean section or LGA was inconclusive; however, the odds of preterm birth (1.94; 1.31, 2.28) and SGA (1.52; 1.18, 1.96) were increased.

**Conclusions:**

Consistently with previous findings, adherence to the IOM recommendations seem to help achieve better pregnancy outcomes. Nevertheless, even in the context of clinical trials, women find it difficult to adhere to them. Further research should focus on identifying ways of achieving a healthier GWG as defined by the IOM recommendations.

**Electronic supplementary material:**

The online version of this article (10.1186/s12884-019-2472-7) contains supplementary material, which is available to authorized users.

## Background

Gestational weight gain (GWG) is a natural response to accommodate the growing fetus. Components of GWG include the body composition (fat, lean mass), the weight of the fetus, placenta, and amniotic fluid [1]. Nonetheless, too high or too low GWG contributes to short- and long-term health complications [2–5], especially when a woman enters pregnancy with a Body Mass Index (BMI) of 25 or above [6–11]. The number of women entering pregnancy with high BMI is increasing [12]. High weight gain in pregnancy occurs in both high-income [13–15] and low-income countries [16, 17]. The US-based Institute of Medicine (IOM), among others, has attempted to identify an optimal amount of GWG [1, 2, 18–20] and has issued recommendations to support healthcare providers advising women on a healthy amount of weight gain in pregnancy [20]. Despite their intention, only marginal improvement in the amount of GWG in the US has been observed [21]. Outside the US, the adoption of the recommendations vary [22]. For example, the UK National Institute for Health and Care Excellence (NICE) did not from endorse the IOM recommendations, considering the evidence base insufficient to guide clinical practice (retrospective population-based cohorts) [22, 23].

Weight gain outside of the IOM recommendations is widespread. In a recent meta-analysis of observational studies with over a million pregnancies, two-thirds of evaluated women gained weight outside the IOM recommendations [24]. As Individual Participant Data (IPD) from those studies was not available, the degree of departure from the recommendations is unknown. Although the meta-analysis reaffirmed the association between GWG outside the IOM recommendations and adverse pregnancy outcomes [4, 10, 17, 24–31], the findings were limited by a lack of adjustment for potential confounders (e.g. gestational age in the analysis for preterm birth), inconsistency in outcome definitions (e.g. of preterm birth). There was also considerable between-study heterogeneity; with a I^2^ value of below 30% in only one analysis (caesarean section and gestational weight gain above the IOM recommendation) in comparison to five analyses where it was 70% or more [24]. Hence, the magnitude of the association, commonly reported for any women whose GWG is above or below the IOM recommendations, is still uncertain. Our work therefore aimed to address these gaps, using a repository of IPD from randomised trials with details of relevant confounders and clear outcome definitions, assembled by the International Weight management in Pregnancy (i-WIP) Collaborative group [32]. For women with GWG outside (above or below) the IOM recommendations we estimated the odds of adverse pregnancy outcomes in comparison to those within (overall and by BMI category), accounting for relevant confounders. We examined the degree to which women departed from the IOM recommended ranges of weight gain, and explored the change in the adjusted odds by the degree of departure.

## Methods

We included studies comprising of pregnant women with a singleton fetus and maternal BMI (pre- or early pregnancy) of 18.5 kg/m^2^ or more, that collected relevant information on GWG. The relevant data were obtained from the i-WIP IPD repository holding data from 36 randomised trials on lifestyle interventions in pregnancy [32, 33] from 16 countries across five geographical regions (North and South America, Europe, Middle East, and Australia) [34]. We only used data from participants allocated to the control arms of those trials (standard antenatal care as defined locally) thereby excluding any potential variation due to intervention effects across the studies. GWG was defined as the difference between the last available antenatal weight (usually around delivery) and the earliest weight measurement during pregnancy or the pre-pregnancy weight if the former was not available [32]. We evaluated both maternal and offspring outcomes, namelycaesarean section (elective or emergency), large for gestational age (LGA) or small for gestational age (SGA) infant, and preterm birth. The outcomes were selected through a formal prioritisation exercise and reflect clinical importance [35]. We harmonised coding of the variables across datasets from all 36 trials [33], coding caesarean delivery as ‘any case of caesarean delivery’ and ‘non-caesarean delivery’; LGA and SGA as growth above the 90th centile, and below the 10th centile respectively; and preterm birth as birth earlier than 37 weeks of gestation. For LGA and SGA we first calculated the birth centiles using gestational age, baby’s birth weight, maternal (pre- or early pregnancy) weight, height and parity [36] before identifying infants with growth above the 90th centile and below the 10th centile.

The total GWG was categorised as above, within or below the IOM recommendations (2009) according to the woman’s initial (early or pre-pregnancy) BMI category as defined by the WHO [37]. The recommended amount of GWG is 11.5–16 kg, 7–11.5 kg, and 5–9 kg for women entering pregnancy with healthy BMI (18.5–24.9 kg/m^2^) - “normal BMI” in the WHO classification [37]; overweight (25–29.9 kg/m^2^) and obese (≥ 30 kg/m^2^) respectively [20]. For women with a total GWG outside (above or below) the IOM recommendations, we calculated the absolute difference between the recorded value and the limit of the recommended GWG and coded the direction of the difference (above or below the IOM recommendations). For example, for a woman with healthy BMI (18.5–24.9 kg/m^2^) where the recommended range is 11.5 to 16 kg, a total GWG of 18 kg was coded as GWG of 2 kg above the IOM recommendations. In the same BMI category, a total GWG of 10 kg was coded as GWG of 1.5 kg below the IOM recommendations.

We identified the potential confounders of the relationship between the exposure (total GWG classified according to the IOM recommendations) and the adverse pregnancy outcomes through a literature review and based on a consultation with the clinical experts (APB, ST). The confounders were prioritised from the clinical perspective, and their availability assessed in the dataset (Additional file 1). The number of covariates per model was limited by the number of events (one covariate per 10 events) to prevent overfitting [38]. Regression models with caesarean section as of outcome were adjusted for occurrence of any diabetes-related event (defined as gestational diabetes or diabetes prior to pregnancy - yes/no), women’s age (continuous), gestational age at delivery (continuous), parity (nullipara/multipara), and smoking status (yes/no). Models with LGA were adjusted for any diabetes-related events (yes/no) and women’s age (continuous), and models with SGA for smoking status (yes/no), women’s age (continuous) and parity (nullipara/multipara). Due to a low number of events, models for preterm birth could only be adjusted for smoking status (yes/no). Moderators in the causal pathways between the exposure and adverse pregnancy outcomes, e.g. LGA for caesarean section, were not taken into account in the adjusted models [38].

### Statistical analysis

The characteristics were summarised as counts and percentages (categorical and dichotomous data), or as means and standard deviations (SD) (continuous data). Firstly, we examined the distribution of total GWG by each kilogram outside (above or below) the IOM recommendations and described it using the median, lower [25] and upper (75) quartiles. The number of women and events were tabulated according to the IOM categories. We examined the relationship of GWG outside (above or below) the IOM recommendations and adverse pregnancy outcomes using a one-stage IPD meta-analytical framework.

In all models, we applied a mixed-effects logistic regression, accounting for clustering of participants within the studies by including random effects for baseline differences on a study level [39]. Firstly, we computed the odds ratio of adverse maternal and offspring outcomes for women with GWG outside (above or below) versus within the IOM recommendations, accounting for relevant confounders. Secondly, we assessed the impact of the magnitude of GWG outside (above or below) the IOM recommendation on the odds of adverse pregnancy outcomes. Due to the skewed distribution of the exposure, we split it into quartiles and computed the odds of adverse outcomes for each quartile of GWG outside (above or below) the IOM recommendations in comparison to within. The main models were performed including all women, irrespective of their (pre- or early pregnancy) BMI, but we accounted for these values in the analysis. We subsequently assessed the effects by BMI category (healthy BMI, overweight and obese). The relationship between the exposure and adverse outcomes was described using odds ratio (OR) with respective 95% confidence intervals (CI). There is no robust methodology to quantify inter-study heterogeneity when using a one-stage random effects model [40]. However, in cluster data analysis the I^2^ is very similar to the intraclass correlation coefficient (ICC) [41] that we calculated for the adjusted models. We did not attempt to impute any missing data. All analyses were performed using Stata (version 14.1) with statistical significance considered at the 5% level and no correction for multiple testing.

A sensitivity analysis was performed for preterm birth models to explore the impact of potential misclassification of women who did not reach full term. An alternative indicator of adherence to the IOM recommendations is by a rate of GWG per week of pregnancy – for women with healthy BMI 0.35–0.50 kg, overweight women 0.23–0.33 kg and obese women 0.17–0.27 kg [20, 42]. The values refer to rate of the GWG in the second and third trimester and assume a linear progression of GWG [20]. Accordingly, we calculated the rate of GWG by dividing the total recorded GWG by the number of completed gestational weeks in those trimesters.

## Results

Individual records of 4429 women across 33 datasets were available for analysis. The majority of women in the available dataset were of Caucasian origin (91.3%), over half were highly educated (55.8%) and in their first pregnancy (51.3%). More than one-third (36.6%) had a healthy BMI (pre- or early pregnancy), and over one-third (35.3%) were obese (BMI ≥ 30 kg/m^2^) (Table 1). The characteristics of women across the IOM categories (above, within, and below) were broadly comparable, with minor differences in the distribution by education classes, smoking status, and presence of any diabetes-related events (Additional file 2).

**Table 1:** Characteristics of women in the control arms of randomised trials included in the analyses

Characteristics	Number of studies (women)	Mean (SD) or Frequency (%)
Age (years)	32 (4415)	30.1 (5.1)
Height (cm)	31 (4422)	165.0 (7.0)
Weight^a^ (kg)	33 (4429)	77.13 (18.4)
Body Mass Index (kg/m^2^)	31 (4429)	28.32 (6.37)
Body Mass Index categories	31 (4429)	
Healthy BMI (BMI 18.5–24.99 kg/m^2^)^b^		1622 (36.6)
Overweight (BMI 25–29.99 kg/m^2^)		1245 (28.1)
Obese (BMI ≥ 30 kg/m^2^)		1562 (35.3)
Ethnic origin	24 (3536)	
Caucasian		3232 (91.3)
Non-Caucasian		304 (8.7)
Education level^c^	27 (3332)	
Basic		453 (13.6)
Intermediate		1019 (30.6)
Higher		1860 (55.8)
Parity	30 (4317)	
0		2113 (49.0)
1+		2204 (51.0)
Current smoker	27 (3964)	693 (16.5)
Inactive before pregnancy^d^	25 (2760)	1377 (50.1)
Family history of diabetes	10 (1784)	455 (26.2)
Hypertension at baseline	20 (2154)	53 (2.5)
Any hypertensive event in pregnancy^e^	24 (3502)	318 (9.1)
Any case of diabetes-related events^f^	31 (4422)	448 (10.1)
Gestational age at delivery (weeks)	31 (4419)	39.6 (1.6)

^a^Early or pre pregnancy weight;

^b^equivalent of Body Mass Index (BMI) termed as normal in the World Health Organization classification [20]

^c^’low’ (secondary education completed before A-levels), ‘medium’ (secondary education to A-level equivalent) or ‘high’ (any further/higher education) for details see Table 48 in Rogozinska et al. 2017 [33]

^d^Defined as no exercise or sedentary lifestyle prior to pregnancy for details see Table 49 in Rogozinska et al. 2017 [33]

^e^Pregnancy Induced Hypertension, high blood pressure, pre-eclampsia;

^f^Gestational Diabetes Mellitus or pre-pregnancy Diabetes Mellitus;

**Fig. 1:**
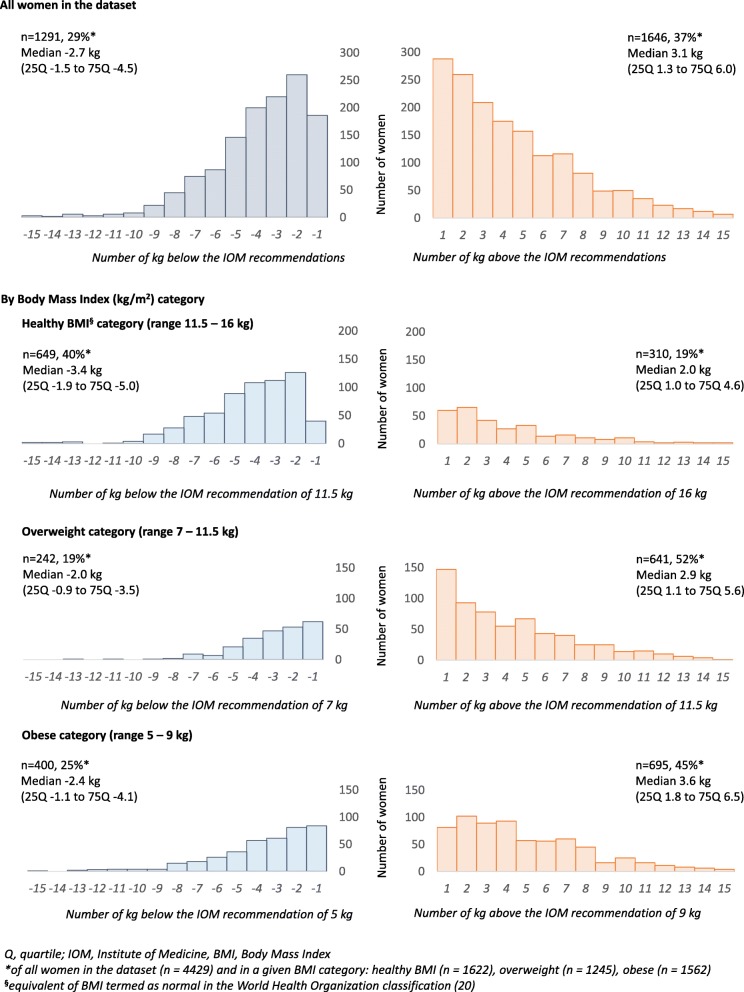
Distribution of kilograms of gestational weight gain outside the Institute of Medicine recommendations (2009)

Two-thirds of women gained weight outside the IOM recommendations, 36.6% (1646/4429) were above, and 29% (1291/4429) were below. Nearly half of the women with GWG above the IOM recommendations (46.9%, 772/1646), the upper limit by one to three kilograms (Fig. 1). Over half of women (52.6%, 678/1291) with GWG below the IOM recommendations were between one to three kilograms below the IOM recommendations (Fig. 1). Weight gain outside (above or below) the IOM recommendations varied between the BMI categories (*p* < 0.001, Pearson Chi^2^). Over half of overweight (641/1646; median GWG outside the IOM recommendations of 2.9 kg) and 45% of obese women (695/1245; median GWG outside the IOM recommendations of 3.6 kg) gained above the IOM recommendations, compared to only 19% in the healthy BMI category (310/1646, median 2.0 kg). GWG was above the IOM recommendations by 1 kg in 20.6% (64/310), 23.6% (151/641), and 11.7% (81/695) of women with a healthy BMI, overweight and obese women respectively (Fig. 1) (Additional file 3). More women with a healthy BMI gained below the IOM recommendations (40%, 649/1291; median − 3.4 kg) in comparison to overweight (19%, 242/1291; median − 2.0 kg) and obese women (25%, 400/1291; median − 2.4 kg). The weight gain was below the IOM recommendations by 1 kg in 6.2% of women with a healthy BMI (40/649), compared to 25.6% (62/242) and 21.3% (85/400) in overweight and obese women (Fig. 1).

### Adverse pregnancy outcomes in women with GWG above the IOM recommendations

**Fig. 2:**
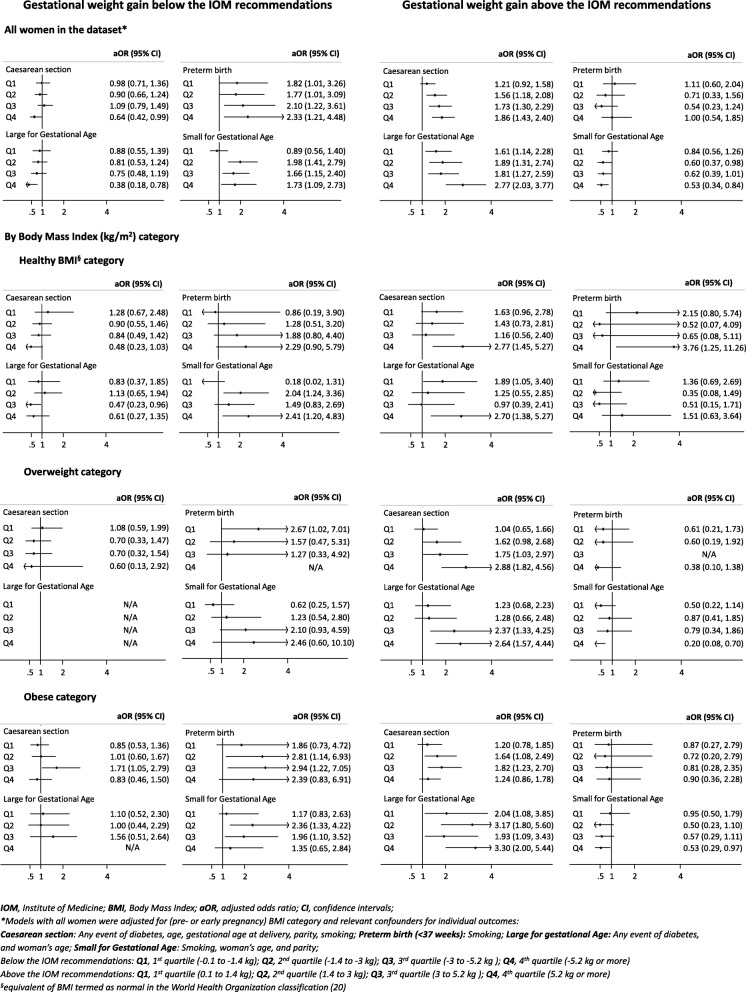
Quartiles of gestational weight gain outside the Institute of Medicine recommendations (2009) and pregnancy complications

Compared to women with GWG within the IOM recommendations, those who gained above had increased odds of caesarean section (aOR 1.50, 95% CI 1.25, 1.80; ICC 0.055) (Table 2). This increase was observed across all baseline BMI categories – healthy BMI (aOR 1.58, 95% CI 1.09, 2.28; ICC 0.053), overweight (aOR 1.68, 95% CI 1.19, 2.35; ICC 0.071) and obese (aOR 1.44, 95% CI 1.10, 1.89; ICC 0.027) (Table 2). The exploration of the effect by quartile of GWG above the IOM recommendations showed an increasing effect with greater GWG departures (Fig. 2). We did not observe an association of GWG above the IOM recommendations with preterm birth (Table 2).

**Table 2:** Gestational weight gain outside versus within the Institute of Medicine recommendations (2009) and the adverse pregnancy outcomes

BMI category	No. studies (women)	OR (95% CI)	No. studies (women)	aOR (95% CI)	No. studies (women)	OR (95% CI)	No. studies (women)	aOR (95% CI)
	Gestational weight gain above the IOM recommendations							
	Caesarean section^a^				Preterm birth^b^			
All women^e^	*30 (2727)*	*1.42 (1.20, 1.68)*	24 (2700)	1.50 (1.25, 1.80)	*30 (3126)*	*0.75 (0.50, 1.11)*	26 (2769)	0.84 (0.54, 1.29)
Healthy BMI^f^ (16 kg)	*21 (949)*	*1.36 (0.96, 1.92)*	21 (781)	1.58 (1.09, 2.28)	*21 (971)*	*1.40 (0.70, 2.80)*	19 (809)	1.73 (0.82, 3.65)
Overweight (11.5 kg)	*29 (982)*	*1.43 (1.04, 1.98)*	23 (877)	1.68 (1.19, 2.35)	*29 (1000)*	*0.32 (0.15, 0.68)*	25 (897)	0.40 (0.18, 0.86)
Obese (9 kg)	*30 (1143)*	*1.29 (1.00, 1.68)*	24 (1042)	1.44 (1.10, 1.89)	*30 (1155)*	*0.81 (0.41, 1.59)*	26 (1063)	0.89 (0.44, 1.80)
	Large for Gestational Age^c^				Small for Gestational Age^d^			
All women^e^	*31 (3138)*	*1.85 (1.47, 2.32)*	30 (3123)	2.00 (1.58, 2.54)	*30 (3123)*	*0.68 (0.52, 0.87)*	25 (2754)	0.66 (0.50, 0.87)
Healthy BMI (16 kg)	*21 (973)*	*1.77 (1.17, 2.70)*	20 (967)	1.68 (1.10, 2.56)	*21 (970)*	*0.89 (0.54, 1.44)*	18 (803)	0.93 (0.56, 1.56)
Overweight (11.5 kg)	*29 (1003)*	*1.68 (1.11, 2.53)*	28 (998)	1.83 (1.20, 2.80)	*29 (1000)*	*0.44 (0.27, 0.74)*	24 (897)	0.51 (0.30, 0.87)
Obese (9 kg)	*31 (1162)*	*2.53 (1.67, 3.83)*	30 (1158)	2.75 (1.80, 4.19)	*30 (1153)*	*0.71 (0.48, 1.05)*	25 (1054)	0.65 (0.42, 0.98)
	Gestational weight gain below the IOM recommendations							
	Caesarean section^a^				Preterm birth^b^			
All women^e^	*30 (3074)*	*0.93 (0.76, 1.13)*	24 (2395)	0.93 (0.75, 1.13)	*30 (2769)*	*1.81 (1.26, 2.59)*	26 (2486)	1.94 (1.31, 2.88)
Healthy BMI (11.5 kg)	*21 (1285)*	*0.84 (0.60, 1.17)*	21 (1082)	0.79 (0.55, 1.14)	*21 (1309)*	*1.69 (0.95, 3.01)*	19 (1131)	1.65 (0.86, 3.17)
Overweight (7 kg)	*29 (590)*	*0.99 (0.65, 1.51)*	23 (536)	0.83 (0.53, 1.31)	*29 (601)*	*1.28 (0.62, 2.64)*	25 (562)	1.58 (0.73, 3.43)
Obese (5 kg)	*30 (852)*	*1.07 (0.80, 1.43)*	24 (777)	1.10 (0.81, 1.51)	*30 (859)*	*2.40 (1.28, 4.50)*	26 (793)	2.39 (1.22, 4.68)
	Large for Gestational Age^c^				Small for Gestational Age^d^			
All women^e^	*31 (2783)*	*0.79 (0.59, 1.05)*	30 (5880)	0.76 (0.57, 1.02)	*30 (2762)*	*1.57 (1.24, 2.00)*	25 (2446)	1.52 (1.18, 1.96)
Healthy BMI (11.5 kg)	*21 (1312)*	*0.77 (0.50, 1.18)*	20 (1294)	0.78 (0.51, 1.20)	*21 (1304)*	*1.71 (1.16, 2.51)*	18 (1113)	1.62 (1.07, 2.45)
Overweight (7 kg)	*29 (604)*	*0.54 (0.28, 1.02)*	28 (599)	0.53 (0.27, 1.02)	*29 (601)*	*1.24 (0.74, 2.09)*	24 (549)	1.24 (0.71, 2.16)
Obese (5 kg)	*31 (467)*	*1.03 (0.62, 1.74)*	30 (864)	0.98 (0.58, 1.66)	*30 (857)*	*1.82 (1.24, 2.66)*	25 (784)	1.81 (1.22, 2.71)

*BMI* Body Mass Index (kg/m^2^), *OR* Odds ratio, *aOR* Adjusted odds ratio, *CI* Confidence intervals, *IOM* Institute of Medicine

Models adjustments ^a^Any event of diabetes, age, gestational age at delivery, parity, smoking; ^b^Smoking; ^c^Any event of diabetes, and woman’s age; ^d^Smoking, woman’s age, and parity; and BMI category; ^e^All relevant confounders and BMI category; statistically significant associations are in bold

Kilogram values in brackets indicate upper (weight gain above) or lower (weight gain below) value of the IOM recommendations (2009) for a given BMI category [20]

^f^equivalent of BMI termed as normal in the World Health Organization classification [20]

Compared to women with GWG within the IOM recommendations, those who gained above the recommendations had increased odds of LGA (aOR 2.00, 95% CI 1.58, 2.54; ICC 0.115). The effect was observed across all baseline BMI categories – healthy BMI (aOR 1.68, 95% CI 1.10, 2.56; ICC 0.103), overweight (aOR 1.83, 95% CI 1.20, 2.80; ICC 0.073) and obese (aOR 2.75, 95% CI 1.80, 4.19; ICC 0.256) (Table 2). Again the effect by quartile of GWG above the IOM recommendations showed an increasing effect with greater GWG departures (Fig. 2). There was a 34% relative decrease in the odds of SGA overall (aOR 0.66, 95% CI 0.50, 0.87; ICC 0.078), with the decrease observed in overweight (aOR 0.51, 95% CI 0.30, 0.87; ICC 0.172) and obese categories (aOR 0.65, 95% CI 0.42, 0.98; ICC not possible to estimate) (Table 2), with an increasing effect observed again with greater departures from the IOM recommendations (Fig. 2).

### Adverse pregnancy outcomes in women with GWG below the IOM recommendations

Compared to women with GWG within the IOM recommendations, for those who gained below the recommendations, we did not observe a statistically significant association with caesarean section (Table 2). The odds of preterm birth were increased by 94% (aOR 1.94, 95% CI 1.25, 1.80; ICC 0.149) with a significant increase observed only in the obese category (aOR 2.39, 95% CI 1.22, 4.68; ICC 0.179) (Table 2). The exploration of the effect by quartile of GWG below the IOM recommendations showed an increasing effect with greater departures (Fig. 2).

Compared to women with GWG within the IOM recommendations, for those who gained below the recommendations, we did not observe a statistically significant association with LGA. The odds of SGA was increased by 52% (aOR 1.52, 95% CI 1.18, 1.96; ICC 0.078) (Table 2). The effect for SGA was observed in healthy BMI (aOR 1.62, 95% CI 1.07, 2.45; ICC 0.141) and obese categories (aOR 1.81, 95% CI 1.22, 2.71; ICC not possible to estimate) (Table 2). We did not observe any clear trend in the analysis by quartile of GWG below the IOM recommendations (Fig. 2).

### Sensitivity analysis

The analysis for preterm birth using the IOM classification based on average weekly weight gain returned effect estimates comparable to those obtained from the models where women were classified based on their total GWG (Additional file 4).

## Discussion

In our dataset comprised of women from the control arms (standard antenatal care) of 33 randomised trials across 16 countries, two-thirds of women gained weight outside the IOM recommendations. The degree of GWG outside the recommendations varied depending on the women’s pre-pregnancy BMI but was commonly up to 3 kg irrespective of the direction (above: median 3.1 kg; below: median − 2.7 kg). GWG above the IOM recommendations was most common in the obese subgroup (median 3.6 kg) while women with healthy BMI (median − 3.4 kg) were most likley to have GWG below the IOM recommendations.

Weight gain outside the IOM recommendations was associated with a change in the odds of adverse pregnancy outcomes. In comparison to weight gain within the IOM recommendations, GWG above the recommended amount was associated with 50% increased odds of caesarean section and a two-fold odds of LGA. Conversely, the odds of SGA were reduced by 36%, and had no conclusive effect on preterm birth. For weight gain below the IOM recommendations, however, the odds of preterm birth was increased almost two-fold and of SGA by 50%. The odds of LGA were decreased by 24%. There was no conclusive effect on the caesarean section rate. The direction of the effects was consistent across BMI category with the odds of an adverse pregnancy outcome being highed for the most extreme departures from the IOM recommendations (5 kg or more).

Our study was conducted using IPD from an international dataset of randomised trials and contributes to the body of evidence on the relationship between amount of gestational weight gain and pregnancy outcomes [34]. The work avoids limitations of previous primary studies evaluating the non-adherence to the IOM recommendations, which were mostly constrained to a specific cohort of women (geographical or BMI limitations), and secondary studies using aggregate study-level data that do not allow for individual level adjustment [10, 24, 28, 29, 43, 44]. Access to IPD in meta-analytical approach allows adjusting for relevant confounders and detecting participant rather than study-level associations – a common limitation of study-level meta-analysis [45, 46]. The adjustment of the models in our analysis had an effect on the magnitude of the pooled estimates. The ICC, which we used to estimate an approximation of between-study heterogeneity, was between 3 and 26%, suggesting reasonable consistency between the studies. Finally, direct contact with trial authors facilitated data integrity checks and allowed standardisation of definitions for outcomes such as LGA, SGA and preterm birth.

There are some limitations to our work. Even though we used data from a cohort of women allocated to control arms (standard antenatal care) of trials targeting change in eating habits or activity level, the participation in the trial on its own could affect women’s behaviour and indirectly impact the amount of gained weight [47, 48].

The ethnicity of the participants in the dataset (over 90% of Caucasian descent) potentially reduces the generalisability of the findings onto other (non-Caucasian) populations. However, there is no strong evidence that the link between GWG and pregnancy complication differs across ethnicities [49], and the evidence base for the IOM recommendations is itself limited as it mostly refers to data from predominantly Caucasian women from developed countries [1, 20].

The complex nature of the dataset with clustering of records within the original trials creates particular challenges. For example, important covariates (e.g. fetal presentation for caesarean section) were not always available in the individual trial datasets which resulted in the statistical models not being adjusted for all relevant confounders. Furthermore, in the analyses, we only used data from women allocated to control arms to simplify the statistical models and improve the clinical interpretability of their findings. This contributed to small samples of participants available for analysis of less frequent outcomes (SGA and preterm birth) and within BMI category (Additional file 5). Secondly, despite access to patient-level records (IPD), some of the encountered limitations were comparable to those reported for other meta-analyses on the subject synthesis [24–26, 28, 29, 50]. For example, we could not use 23% of records in the repository due to lack of initial or follow-up measures (for two trials, data was provided as total GWG instead of individual weight measures). It was also not always possible to use the measurement at the same time point for the initial weight value (use of pre or early pregnancy weight) and ensure the accuracy of its unbiased recording (self-reported versus objectively measured). Moreover, the lack of measurements of weight at the time of diagnosis did not permit exploration of the relationship with outcomes such as pregnancy-induced hypertension, pre-eclampsia or gestational diabetes.

We identified the potential confounders through a non-systematic literature search and prospectively prioritised them from the clinical perspective. The infant’s birth weight was not considered as a potential confounder in any of the models, as it is a component of GWG (examined exposure) and outcomes such as SGA or LGA. In the analyses with the caesarean section as a dependent variable, the infant’s birth weight, especially high birth weight (LGA or macrosomia), was classified as a moderator of the exposure effect (women’s gestational weight gain) on the outcome and therefore not included in the model. The outcomes were selected from a group of maternal and offspring outcomes prioritised for their importance to women’s care in the context of GWG management [35] and were concordant with the outcomes evaluated by the IOM committee when defining optimal GWG [20]. Finally, the findings of our analyses may need to be treated with caution due to the lack of correction for multiple testing.

As has been observed elsewhere [24], the majority of women in our dataset gained outside the IOM recommendations. The IOM recommendations were commonly not met by 0.1 up to 3 kg (above or below), and the direction and magnitude of GWG outside the recommendations varied across the BMI category. More overweight and obese women gained weight above the IOM recommendations than those who entered pregnancy with a healthy BMI. Pregnant women entering pregnancy overweight or obese are a group of particular interest due to the risk of complications being increased [11, 51]. The IOM recommendations incorporate this additional risk by lowering the amount of GWG for those BMI categories in comparison to women with healthy pre-pregnancy BMI [20]. However, the literature consistently shows that women from those BMI categories frequently struggle to gain weight within the recommended ranges [13, 27, 52] and carry over extra weight into subsequent pregnancies [53].

The direction of the pooled effects in the adjusted analyses was mostly consistent with previous reports [24, 28, 29]. The exploratory analyses by quartile of weight gain outside (above or below) the IOM recommendations showed larger effects for the gain in the fourth quartile (5 kg or more), and were frequently inconclusive for the first (0.1 to 1.4 kg) and second quartiles (1.4 to 3 kg). This may be due to insufficient sample size in our dataset (especially for preterm birth) or beacause of a weaker effect of smaller amounts of weight gain outside the IOM recommendations (0.1 to 1.4 kg). Nevertheless, a dose-response effect of weight gain was clearly observed for caesarean section, LGA and SGA and GWG above the IOM recommendations.

The prevention of excessive weight gain in pregnancy is one of the WHO priorities for achieving a positive pregnancy experience [54]. Regular monitoring of weight gain in pregnancy and provision of specific recommendations are at present not part of standard antenatal care in the United Kingdom [23] nor many other developed countries. Although the IOM recommendations are widely disseminated and evaluated in clinical studies, the amount of GWG they recommend was derived from a predominantly Caucasian population, and their use in ethnically diverse populations may not accurately describe the relationship between low or high GWG and its adverse pregnancy outcomes [55]. The distribution of GWG outside the IOM recommendations needs to be explored in a large, ethnically diverse prospective population-based study to confirm or refute our observations. Taking into account the rise of caesarean section rates [56] and increased weight gain in pregnancy [12], future studies should explore their relationship in more detail. Moreover, it is crucial to assemble a dataset that will allow exploration of the relationship of weight gain in pregnancy with other important outcomes that could not be explored in our study, especially gestational diabetes [57].

## Conclusions

Consistently with previous findings, adherence to the IOM recommendations seems to help achieve better pregnancy outcomes. Even a moderate amount of GWG outside the IOM recommendations adjusted for relevant characteristics was associated with an increased risk of negative maternal and offspring outcomes. Nevertheless, even in the context of clinical trials, women find it challenging to meet the IOM recommended amount of healthy GWG. Further research should focus on identifying ways of achieving a healthier GWG as defined by the IOM recommendations.

## Additional files

Additional file 1: Lists of potential confounders. Tables with confounders considered for individual models depending on the outcome of interest (DOCX 20 kb)

Additional file 2: Characteristics of women classified according to the Institute of Medicine recommendations (2009). Table with baseline characteristics of women from the control arms of randomised trials used in the analyses classified by adherence to the Institute of Medicine (2009) recommendations (DOCX 21 kb)

Additional file 3: Proportion of women with gestational weight gain outside the Institute of Medicine recommendations (2009) by kilogram. Number and proportion of women by each kilogram of GWG above (A) or below (B) the Institute of Medicine recommendations (2009) - overall and by baseline BMI category (DOCX 19 kb)

Additional file 4: Sensitivity analyses for preterm delivery using classification of gestational weight gain by week. Summary of sensitivity analyses of a relationship between gestational weight gain outside (above or below) versus within the Institute of Medicine recommendations (2009) and preterm birth using classification based on weekly weight gain (DOCX 16 kb)

Additional file 5: Adverse pregnancy outcomes according to adherence to the Institute of Medicine recommendations (2009). Proportion of adverse pregnancy outcomes according to adherence to the Institute of Medicine recommendations (2009) overall and by baseline BMI category (DOCX 18 kb)

## Data Availability

The full dataset or its subset and technical appendix are available from the data custodian (Queen Mary University of London) at smd-iwipdata@qmul.ac.uk. Access to the dataset is regulated by terms and conditions available on request. The presented data are anonymised, and risk of identification of individual participants is low.

## Abbreviations

BMI: Body Mass Index
GWG: Gestational weight gain
IOM: Institute of Medicine
