# Supplementary material for: Gestational weight gain outside the Institute of Medicine recommendations and adverse pregnancy outcomes: analysis using individual participant data from randomised trials
Source: BMC Pregnancy Childbirth. 2019 Sep 2;19:322. doi: 10.1186/s12884-019-2472-7 (PMC6719382; doi:10.1186/s12884-019-2472-7)
Supplement: Supplementary file 1 — Lists of potential confounders. Tables with confounders considered for individual models depending on the outcome of interest (DOCX 20 kb) [file 12884_2019_2472_MOESM1_ESM.docx]

Additional file 1. Lists of potential confounders

1. Outcome: any type of caesarean section

| **Considered confounders** | **Remarks** |
| --- | --- |
| - Booking BMI (kg/m2) | Stratification factor |
| - Diabetes prior to pregnancy or in pregnancy | Mandatory confounder |
| - Age | Potential confounder (1) |
| - Gestational age at delivery | Potential confounder (2) |
| - Parity | Potential confounder (3) |
| - Smoking | Potential confounder (4) |
| - Education level | Potential confounder (5) |
| - Ethnic origin | Potential confounder (6) |
| - Exercise prior to pregnancy | Potential confounder (7) |
| - Pre‑existing vascular disease such as hypertension | Potential confounder (8) Available as ‘Baseline hypertension’ |
| - Induction of labour | Potential confounder (9) |
| - Multiple pregnancy | Only singletons in the dataset |
| - Pregnancy interval of more than 10 years | *Information not available in the dataset* |
| - Family history of pre‑eclampsia | *Information not available in the dataset* |
| - Previous history of pre‑eclampsia | *Information not available in the dataset* |
| - Pre‑existing renal disease | *Information not available in the dataset* |
| - Previous macrosomia | *Information not available in the dataset* |

1. Outcome: Large for Gestational Age (LGA) infant

| **Considered confounders** | **Remarks** |
| --- | --- |
| - Booking BMI (kg/m2) | Stratification factor |
| - Diabetes prior to pregnancy or in pregnancy | Mandatory confounder, available as any diabetes related event |
| - Age | Potential confounder (1) |
| - Parity | Potential confounder (2) |
| - Smoking | Potential confounder (3) |
| - Education level | Potential confounder (4) |
| - Ethnic origin | Potential confounder (5) |
| - Exercise prior to pregnancy | Potential confounder (6) |
| - Pre‑existing vascular disease such as hypertension | Potential confounder (7), baseline hypertension |
| - Multiple pregnancy | Dataset with singleton pregnancy only |
| - Previous macrosomia | *Low availability in the dataset* |
| - Pregnancy interval of more than 10 years | *Information not available in the dataset* |
| - Family history of pre‑eclampsia | *Information not available in the dataset* |
| - Previous history of pre‑eclampsia | *Information not available in the dataset* |
| - Pre‑existing renal disease | *Information not available in the dataset* |

1. Outcome: Small for Gestational Age (SGA) infant

| **Considered confounders** | **Remarks** |
| --- | --- |
| - Booking BMI (kg/m^2^) | Stratification factor |
| - Smoking | Mandatory confounder |
| - Age | Potential confounder (1) |
| - Parity | Potential confounder (2) |
| - Education level | Potential confounder (3) |
| - Ethnic origin | Potential confounder (4) |
| - Exercise prior to pregnancy | Potential confounder (5) |
| - Pre‑existing vascular disease such as hypertension | Potential confounder (6) |

1. Outcome: preterm delivery (before 37 week of gestation)

| **Considered confounders** | **Remarks** |
| --- | --- |
| - Booking BMI (kg/m^2^) | Stratification factor |
| - Smoking | Mandatory confounder |
| - Age | Potential confounder (1) |
| - Diabetes prior to pregnancy or in pregnancy | Potential confounder (2) |
| - Parity | Potential confounder (3) |
| - High blood pressure | Potential confounder (4) Available as any hypertensive disease in pregnancy |
| - Education level | Potential confounder (5) Used as a proxy of socioeconomic status |
| - Ethnic origin | Potential confounder (6) |
| - Exercise prior to pregnancy | Potential confounder (6) |
| - Multiple pregnancy | Only singletons in the dataset |
