# Supplementary material for: Gestational weight gain outside the Institute of Medicine recommendations and adverse pregnancy outcomes: analysis using individual participant data from randomised trials
Source: BMC Pregnancy Childbirth. 2019 Sep 2;19:322. doi: 10.1186/s12884-019-2472-7 (PMC6719382; doi:10.1186/s12884-019-2472-7)
Supplement: Supplementary file 2 — Characteristics of women classified according to the Institute of Medicine recommendations (2009). Table with baseline characteristics of women from the control arms of randomised trials used in the analyses classified by adherence to the Institute of Medicine (2009) recommendations (DOCX 21 kb) [file 12884_2019_2472_MOESM2_ESM.docx]

Additional file 2. Characteristics of women classified according to the Institute of Medicine recommendations (2009)

| **Characteristics** | **Below the IOM recommendations** | | **Within the IOM recommendations** | | **Above the IOM recommendations** | |
| --- | --- | --- | --- | --- | --- | --- |
|  | **Number of studies (women)** | **Mean (SD) or Frequency (%)** | **Number of studies (women)** | **Mean (SD) or Frequency (%)** | **Number of studies (women)** | **Mean (SD) or Frequency (%)** |
| Age (years) | 31 (1282) | 30.1 (5.3) | 31 (1489) | 30.1 (5.1) | 30 (1644) | 29.7 (5.0) |
| Height (cm) | 31 (1290) | 164.9 (7.3) | 31 (1487) | 164.8 (6.9) | 30 (1645) | 165.1 (6.8) |
| Weight^1^ (kg) | 31 (1291) | 76.0 (20.3) | 31 (1492) | 74.7 (18.0) | 30 (1646) | 80.3 (16.8) |
| Body Mass Index (kg/m^2^) | 31 (1291) | 27.9 (7.2) | 31 (1492) | 27.5 (6.2) | 30 (1646) | 29.4 (5.7) |
| Body Mass Index categories | 31 (1291) |  | 31 (1492) |  | 30 (1646) |  |
| *Healthy BMI (BMI 18.5-24.99 kg/m^2^)^2^* |  | *649 (50.3)* |  | *663 (44.4)* |  | *310 (18.8)* |
| *Overweight (BMI 25-29.99 kg/m^2^)* |  | *242 (18.8)* |  | *362 (24.3)* |  | *641 (38.9)* |
| *Obese (BMI ≥ 30 kg/m^2^)* |  | *400 (30.9)* |  | *467 (31.3)* |  | *695 (42.3)* |
| Ethnic origin | 23 (1013) |  | 23 (1193) |  | 22 (1319) |  |
| *Caucasian* |  | *904 (89.2)* |  | *1096 (91.9)* |  | *1222 (92.6)* |
| *Non-Caucasian* |  | *109 (10.8)* |  | *97 (8.1)* |  | *97 (7.4)* |
| Education level^3^ | 26 (1030) |  | 26 (1153) |  | 26 (1140) |  |
| *Basic* |  | *103 (10.0)* |  | *156 (13.5)* |  | *194 (17.0)* |
| *Intermediate* |  | *299 (29.0)* |  | *343 (29.8)* |  | *372 (32.6)* |
| *Higher* |  | *628 (61.0)* |  | *654 (56.7)* |  | *574 (50.4)* |
| Parity | 29 (1258) |  | 29 (1458) |  | 28 (1592) |  |
| *0* |  | *551 (43.8)* |  | *735 (50.4)* |  | *824 (51.8)* |
| *1+* |  | *707 (56.2)* |  | *723 (49.6)* |  | *768 (48.2)* |
| Current smoker | 27 (1181) | 182 (15.4) | 27 (1318) | 230 (17.5) | 26 (1461) | 280 (19.2) |
| Inactive before pregnancy^4^ | 24 (850) | 420 (49.4) | 24 (962) | 503 (52.3) | 23 (940) | 450 (47.9) |
| Family history of diabetes | 10 (592) | 135 (22.8) | 10 (531) | 135 (25.4) | 10 (661) | 185 (28.0) |
| Hypertension at baseline | 19 (743) | 14 (1.9) | 19 (668) | 16 (2.4) | 19 (732) | 16 (2.2) |
| Any hypertensive event in pregnancy^5^ | 24 (1044) | 76 (7.3) | 24 (1160) | 88 (7.6) | 24 (1298) | 154 (11.9) |
| Any case of diabetes-related events^6^ | 30 (1283) | 172 (13.4) | 30 (1486) | 142 (9.6) | 29 (1642) | 134 (8.2) |
| Gestational age at delivery (weeks) | 30 (1282) | 39.5 (1.8) | 30 (1483) | 39.7 (1.5) | 30 (1643) | 39.7 (1.5) |

*IOM, Institute of Medicine*

*^1^Early or pre pregnancy weight;*

*^2^equivalent of BMI termed as normal in the World Health Organization classification (20)*

*^3^’low’ (secondary education completed before A-levels), ‘medium’ (secondary education to A-level equivalent) or ‘high’ (any further/higher education) for details see Table 48 in Rogozinska et al. 2017 (33)*

*^4^Defined as no exercise or sedentary lifestyle prior to pregnancy for details see Table 49 in Rogozinska et al. 2017 (33)*

*^5^Pregnancy Induced Hypertension, high blood pressure, pre-eclampsia;*

*^6^ Gestational Diabetes Mellitus or pre-pregnancy Diabetes Mellitus;*
