# Supplementary material for: Gestational weight gain outside the Institute of Medicine recommendations and adverse pregnancy outcomes: analysis using individual participant data from randomised trials
Source: BMC Pregnancy Childbirth. 2019 Sep 2;19:322. doi: 10.1186/s12884-019-2472-7 (PMC6719382; doi:10.1186/s12884-019-2472-7)
Supplement: Supplementary file 3 — Proportion of women with gestational weight gain outside the Institute of Medicine recommendations (2009) by kilogram. Number and proportion of women by each kilogram of GWG above (A) or below (B) the Institute of Medicine recommendations (2009) - overall and by baseline BMI category (DOCX 19 kb) [file 12884_2019_2472_MOESM3_ESM.docx]

Additional file 3 Proportion of women with gestational weight gain outside the IOM recommendations (2009) by kilogram

1. Gestational weight gain above the IOM recommendations

| **Above**  **the IOM range (Kg)** | **N of all women** | **%** | **Cum N** | **Cum %** | **N of women with healthy BMI** | **%** | **Cum N** | **Cum %** | **N of overweight women** | **%** | **Cum N** | **Cum %** | **N of obese women** | **%** | **Cum N** | **Cum %** |
| --- | --- | --- | --- | --- | --- | --- | --- | --- | --- | --- | --- | --- | --- | --- | --- | --- |
| 1 | 296 | *18.0%* | 296 | *18.0%* | 64 | *20.6%* | 64 | *20.6%* | 151 | *23.6%* | 151 | *23.6%* | 81 | *11.7%* | 81 | *11.7%* |
| 2 | 264 | *16.0%* | 560 | *34.0%* | 69 | *22.3%* | 133 | *42.9%* | 93 | *14.5%* | 244 | *38.1%* | 102 | *14.7%* | 183 | *26.3%* |
|  |  |  |  |  |  |  |  |  |  |  |  |  |  |  |  |  |
| 3 | 212 | *12.9%* | 772 | *46.9%* | 42 | *13.5%* | 175 | *56.5%* | 81 | *12.6%* | 325 | *50.7%* | 89 | *12.8%* | 272 | *39.1%* |
| 4 | 177 | *10.8%* | 949 | *57.7%* | 27 | *8.7%* | 202 | *65.2%* | 55 | *8.6%* | 380 | *59.3%* | 95 | *13.7%* | 367 | *52.8%* |
| 5 | 159 | *9.7%* | 1108 | *67.3%* | 33 | *10.6%* | 235 | *75.8%* | 69 | *10.8%* | 449 | *70.0%* | 57 | *8.2%* | 424 | *61.0%* |
| 6 | 115 | *7.0%* | 1223 | *74.3%* | 14 | *4.5%* | 249 | *80.3%* | 44 | *6.9%* | 493 | *76.9%* | 57 | *8.2%* | 481 | *69.2%* |
| 7 | 116 | *7.0%* | 1339 | *81.3%* | 16 | *5.2%* | 265 | *85.5%* | 40 | *6.2%* | 533 | *83.2%* | 60 | *8.6%* | 541 | *77.8%* |
| 8 | 82 | *5.0%* | 1421 | *86.3%* | 12 | *3.9%* | 277 | *89.4%* | 25 | *3.9%* | 558 | *87.1%* | 45 | *6.5%* | 586 | *84.3%* |
| 9 | 49 | *3.0%* | 1470 | *89.3%* | 8 | *2.6%* | 285 | *91.9%* | 25 | *3.9%* | 583 | *91.0%* | 16 | *2.3%* | 602 | *86.6%* |
| 10 | 53 | *3.2%* | 1523 | *92.5%* | 12 | *3.9%* | 297 | *95.8%* | 14 | *2.2%* | 597 | *93.1%* | 27 | *3.9%* | 629 | *90.5%* |
| 11 | 35 | *2.1%* | 1558 | *94.7%* | 4 | *1.3%* | 301 | *97.1%* | 15 | *2.3%* | 612 | *95.5%* | 16 | *2.3%* | 645 | *92.8%* |
| 12 | 24 | *1.5%* | 1582 | *96.1%* | 2 | *0.6%* | 303 | *97.7%* | 10 | *1.6%* | 622 | *97.0%* | 12 | *1.7%* | 657 | *94.5%* |
| 13 | 18 | *1.1%* | 1600 | *97.2%* | 3 | *1.0%* | 306 | *98.7%* | 6 | *0.9%* | 628 | *98.0%* | 9 | *1.3%* | 666 | *95.8%* |
| 14 | 12 | *0.7%* | 1612 | *97.9%* | 2 | *0.6%* | 308 | *99.4%* | 4 | *0.6%* | 632 | *98.6%* | 6 | *0.9%* | 672 | *96.7%* |
| ≥15 | 34 | *2.1%* | 1646 | *100.0%* | 2 | *0.6%* | 310 | *100.0%* | 9 | *1.4%* | 641 | *100.0%* | 23 | *3.3%* | 695 | *100.0%* |
| **Total** | **1646** | **100.0%** |  |  | **310** | **100.0%** |  |  | **641** | **100.0%** |  |  | **695** | **100.0%** |  |  |

IOM, Institute of Medicine; Cum, cumulative, N, number

1. Gestational weight gain below the IOM recommendations

| **Below IOM** | **N of all women** | **%** | **Cum N** | **Cum %** | **N of women with healthy BMI** | **%** | **Cum N** | **Cum %** | **N of overweight women** | **%** | **Cum N** | **Cum %** | **N of obese women** | **%** | **Cum N** | **Cum %** |
| --- | --- | --- | --- | --- | --- | --- | --- | --- | --- | --- | --- | --- | --- | --- | --- | --- |
| -1 | 187 | 14.5% | 187 | 14.5% | **40** | **6.2%** | 40 | 6.2% | **62** | **25.6%** | 62 | 25.6% | **85** | **21.3%** | 85 | 21.3% |
| -2 | 267 | 20.7% | 454 | 35.2% | 129 | **19.9%** | 169 | 26.0% | 56 | **23.1%** | 118 | 48.8% | 82 | **20.5%** | 167 | 41.8% |
|  |  |  |  |  |  |  |  |  |  |  |  |  |  |  |  |  |
| -3 | 224 | 17.4% | 678 | 52.5% | 115 | **17.7%** | 284 | 43.8% | 47 | **19.4%** | 165 | 68.2% | 62 | **15.5%** | 229 | 57.3% |
| -4 | 204 | 15.8% | 882 | 68.3% | 111 | 17.1% | 395 | 60.9% | 35 | 14.5% | 200 | 82.6% | 58 | 14.5% | 287 | 71.8% |
| -5 | 147 | 11.4% | 1029 | 79.7% | 90 | 13.9% | 485 | 74.7% | 21 | 8.7% | 221 | 91.3% | 36 | 9.0% | 323 | 80.8% |
| -6 | 88 | 6.8% | 1117 | 86.5% | 55 | 8.5% | 540 | 83.2% | 7 | 2.9% | 228 | 94.2% | 26 | 6.5% | 349 | 87.3% |
| -7 | 75 | 5.8% | 1192 | 92.3% | 48 | 7.4% | 588 | 90.6% | 9 | 3.7% | 237 | 97.9% | 18 | 4.5% | 367 | 91.8% |
| -8 | 46 | 3.6% | 1238 | 95.9% | 29 | 4.5% | 617 | 95.1% | 2 | 0.8% | 239 | 98.8% | 15 | 3.8% | 382 | 95.5% |
| -9 | 22 | 1.7% | 1260 | 97.6% | 17 | 2.6% | 634 | 97.7% | 1 | 0.4% | 240 | 99.2% | 4 | 1.0% | 386 | 96.5% |
| -10 | 9 | 0.7% | 1269 | 98.3% | 5 | 0.8% | 639 | 98.5% |  | 0.0% | 240 | 99.2% | 4 | 1.0% | 390 | 97.5% |
| -11 | 6 | 0.5% | 1275 | 98.8% | 1 | 0.2% | 640 | 98.6% | 1 | 0.4% | 241 | 99.6% | 4 | 1.0% | 394 | 98.5% |
| -12 | 3 | 0.2% | 1278 | 99.0% |  | 0.0% | 640 | 98.6% |  | 0.0% | 241 | 99.6% | 3 | 0.8% | 397 | 99.3% |
| -13 | 6 | 0.5% | 1284 | 99.5% | 3 | 0.5% | 643 | 99.1% | 1 | 0.4% | 242 | 100.0% | 2 | 0.5% | 399 | 99.8% |
| -14 | 2 | 0.2% | 1286 | 99.6% | 2 | 0.3% | 645 | 99.4% |  | 0.0% | 242 | 100.0% |  | 0.0% | 399 | 99.8% |
| ≤-15 | 5 | 0.4% | 1291 | 100.0% | 4 | 0.6% | 649 | 100.0% |  | 0.0% | 242 | 100.0% | 1 | 0.3% | 400 | 100.0% |
| **Total** | 1291 | 100.0% |  |  | 649 | 100.0% |  |  | 242 | 100.0% |  |  | 400 | 100.0% |  |  |

IOM, Institute of Medicine; Cum, cumulative, N, number
