# Supplementary material for: Gestational weight gain outside the Institute of Medicine recommendations and adverse pregnancy outcomes: analysis using individual participant data from randomised trials
Source: BMC Pregnancy Childbirth. 2019 Sep 2;19:322. doi: 10.1186/s12884-019-2472-7 (PMC6719382; doi:10.1186/s12884-019-2472-7)
Supplement: Supplementary file 4 — Sensitivity analyses for preterm delivery using classification of gestational weight gain by week. Summary of sensitivity analyses of a relationship between gestational weight gain outside (above or below) versus within the Institute of Medicine recommendations (2009) and preterm birth using classification based on weekly weight gain (DOCX 16 kb) [file 12884_2019_2472_MOESM4_ESM.docx]

Additional file 4. Sensitivity analyses for preterm delivery using classification of gestational weight gain by week

| **Gestational weight gain** | **BMI category** | **Classification based on total GWG** | **Classification based on by week GWG** |
| --- | --- | --- | --- |
|  |  | **aOR^1^ (95% CI)** | |
| **Above the IOM recommendations** | All women^2^ | 0.84 (0.54, 1.29) | 1.24 (0.79, 1.96) |
|  | Healthy BMI (16 kg) | 1.73 (0.82, 3.65) | 1.84 (0.96, 3.54) |
|  | Overweight (11.5 kg) | 0.40 (0.18, 0.86) | 0.62 (0.25, 1.53) |
|  | Obese (9 kg) | 0.89 (0.44, 1.80) | 1.04 (0.45, 2.40) |
| **Below the IOM recommendations** | All women^2^ | 1.94 (1.31, 2.88) | 1.79 (1.03, 3.10) |
|  | Healthy BMI (11.5 kg) | 1.65 (0.86, 3.17) | 1.69 (0.70, 4.09) |
|  | Overweight (7 kg) | 1.58 (0.73, 3.43) | 0.43 (0.08, 2.23) |
|  | Obese (5 kg) | 2.39 (1.22, 4.68) | 2.31 (0.94, 5.67) |

*BMI, Body Mass Index (kg/m^2^); aOR, adjusted odds ratio; CI, confidence intervals;*

*Models adjustments 1. Smoking; 2. Smoking and BMI category;*

*Kilogram values in brackets indicate upper (weight gain above) or lower (weight gain below) value of the Institute of Medicine recommended (2009) range for a given BMI subgroup (20)*
