# Supplementary material for: Gestational weight gain outside the Institute of Medicine recommendations and adverse pregnancy outcomes: analysis using individual participant data from randomised trials
Source: BMC Pregnancy Childbirth. 2019 Sep 2;19:322. doi: 10.1186/s12884-019-2472-7 (PMC6719382; doi:10.1186/s12884-019-2472-7)
Supplement: Supplementary file 5 — Adverse pregnancy outcomes according to adherence to the Institute of Medicine recommendations (2009). Proportion of adverse pregnancy outcomes according to adherence to the Institute of Medicine recommendations (2009) overall and by baseline BMI category (DOCX 18 kb) [file 12884_2019_2472_MOESM5_ESM.docx]

Additional file 5. Adverse pregnancy outcomes according to adherence to the Institute of Medicine recommendations (2009)

| **Outcome**  BMI category | **Number of studies (women)** | **Below  the IOM recommendations**  **Event/Total (%)** | **Within  the IOM recommendations**  **Event/Total (%)** | **Above  the IOM recommendations**  **Event/Total (%)** |
| --- | --- | --- | --- | --- |
| **Caesarean section** |  |  |  |  |
| All women | 30 (4345) | 277/1271 (21.8) | 340/1456 (23.4) | 503/1618 (31.1) |
| Healthy BMI | 21 (1585) | 83/636 (13.1) | 112/649 (17.3) | 68/300 (22.7) |
| Overweight | 29 (1221) | 54/239 (22.6) | 76/351 (21.7) | 174/631 (27.6) |
| Obese | 30 (1539) | 140/396 (21.8) | 152/456 (33.3) | 261/687 (38.0) |
| **Preterm birth** |  |  |  |  |
| All women | 30 (4412) | 81/1286 (6.3) | 57/1483 (3.8) | 49/1643 (3.0) |
| Healthy BMI | 21 (1618) | 34/647 (5.3) | 22/662 (3.3) | 14/309 (4.5) |
| Overweight | 29 (1241) | 15/241 (6.2) | 19/360 (5.3) | 13/640 (2.0) |
| Obese | 30 (1553) | 32/398 (8.0) | 16/461 (3.5) | 22/694 (3.2) |
| **Large for gestational age** |  |  |  |  |
| All women | 31 (4429) | 92/1291 (7.1) | 135/1492 (9.1) | 267/1646 (16.2) |
| Healthy BMI | 21 (1622) | 48/649 (7.4) | 62/663 (9.4) | 49/310 (15.8) |
| Overweight | 29 (1245) | 14/242 (5.8) | 37/362 (10.2) | 104/641 (16.2) |
| Obese | 31 (1562) | 30/400 (7.5) | 36/467 (7.7) | 114/695 (16.4) |
| **Small for gestational age** |  |  |  |  |
| All women | 30 (4403) | 186/1280 (14.5) | 157/1482 (10.6) | 117/1641 (7.1) |
| Healthy BMI | 21 (1612) | 76/642 (11.8) | 64/662 (9.7) | 26/308 (8.4) |
| Overweight | 29 (1241) | 33/241 (13.7) | 39/360 (10.8) | 31/640 (4.8) |
| Obese | 30 (1550) | 77/397 (19.4) | 54/460 (11.7) | 60/693 (8.7) |

*BMI, Body Mass Index; IOM, Institute of Medicine;*
